# High-Spin-Locked Fe(II) Spin-Crossover Compounds: THz-EPR Investigations and Spin–Phonon Coupling

**DOI:** 10.1021/acs.inorgchem.6c04101

**Published:** 2026-10-14

**Authors:** Tarek Al Said, Eike Kuhlemann, Haowei Chen, Maurice van Gastel, Marcel Walter, Malte Laurin Schwank, Sangeeta Thakur, Karsten Holldack, Wolfgang Kuch, Felix Tuczek, Frank Neese

**Affiliations:** † Helmholtz-Zentrum Berlin für Materialien und Energie GmbH, Hahn-Meitner-Platz 1, 14109 Berlin, Germany; ‡ Institut für Anorganische Chemie, Christian-Albrechts Universität zu Kiel, Max-Eyth-Strasse 2, 24118 Kiel, Germany; § Department of Molecular Theory and Spectroscopy, Max-Planck-Institut für Kohlenforschung, Kaiser-Wilhelm-Platz 1, 45470 Mülheim an der Ruhr, Germany; ∥ Institut für Experimentalphysik, Freie Universität Berlin, Arnimallee 14, 14195 Berlin-Dahlem, Germany

## Abstract

The development of spin-crossover compounds (SCO) and their modification toward suitable applications demands detailed and intimate knowledge of both their electronic structure and intermolecular interactions within the solid state. To acquire this information, frequency-domain Fourier-transform THz electron paramagnetic resonance (FD-FT THz-EPR) spectroscopy is used to trace magnetic transitions of two SCO systems that retain in the high-spin (*S* = 2) state at low temperatures. Two sets of transitions are observed in the range of 5 to 10 and 30 cm^–1^ at zero-field, which can be explained by zero-field splitting and rhombicity. To adequately simulate the dispersion of these transitions with an applied magnetic field, inclusion of spin–phonon coupling is necessary for one of the compounds.

## Graphical abstract

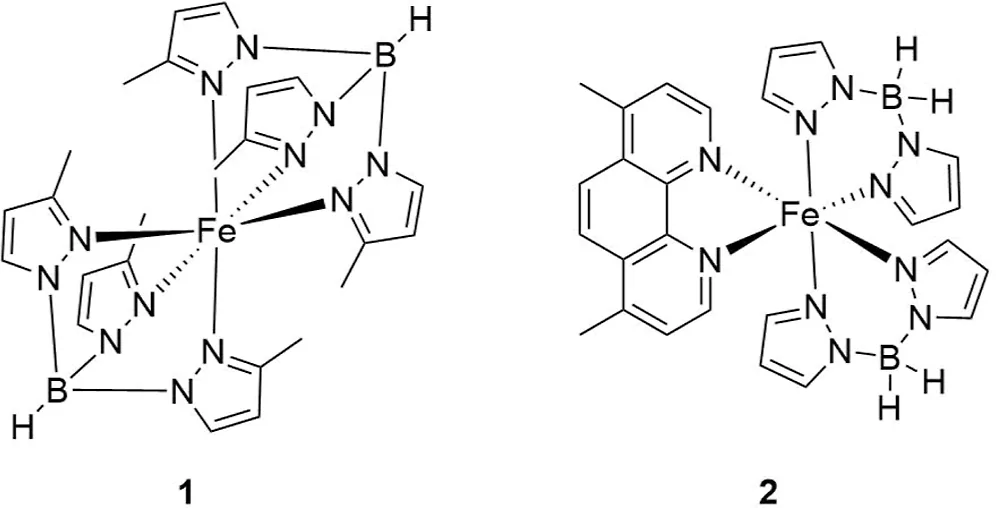

## Introduction

Transition-metal complexes with switchable spin states are of potential interest in molecular electronics and applications, such as data storage or magnetic sensing. The most attractive candidates in this regard are Fe­(II) complexes due to the large difference in bond lengths between the low- and high-spin configurations. Moreover, the two spin states (*S* = 0; low spin (LS) and *S* = 2, high spin (HS)) exhibit markedly different physicochemical properties, and the spin transitions in these systems can be induced by a range of stimuli such as temperature, pressure, or light.^1^ In Fe­(II) systems exhibiting thermal spin transitions, the HS state is typically populated at room temperature and the LS state at low temperature, the latter often allowing excitation to a metastable HS state by light irradiation (light-induced excited spin state trapping effect (LIESST)) at low temperatures.^2^ Targeted optimization of the switching properties of Fe­(II) complexes requires an intimate knowledge of their geometric and electronic structures. In this background, a wide range of spectroscopic and analytical methods can be applied to these systems.^1,2^ More recent techniques in this regard are frequency-domain Fourier-transform THz electron paramagnetic resonance (FD-FT THz-EPR) spectroscopy^3^ or far-infrared magnetospectroscopy (FIRMS).^4^ Essentially, both techniques involve the use of far-IR/THz radiation in conjunction with applied magnetic fields. In contrast to FIRMS, in FD-FT THz-EPR, which is used in this study, the low-energy limit of the spectral window is extended to sub-THz frequencies due to the use of coherent synchrotron radiation.

Recently, we have applied THz and FIR spectroscopy in the absence of magnetic fields to obtain vibrational spectra on the HS–HS, HS–LS and LS–LS forms of a spin-crossover dimer.^5^ Moreover, the THz/FIR window is particularly interesting as magnetic-dipole-allowed transitions between the magnetically split levels of HS Fe­(II) complexes are typically occurring in the THz energy range.^6–10^ Notably, FD-FT THz-EPR allows us to distinguish these transitions from vibrational transitions due to their field-dependency. This renders FD-FT THz-EPR spectroscopy a unique and powerful tool to obtain accurate information on the electronic structure of paramagnetic molecules. Nevertheless, this information is not always easy to extract from the experimental data, and advanced theoretical methods allowing a full understanding of THz spectra, including the effects of spin–phonon coupling, are still under development.^11,12^

Herein, in order to illuminate electronic, magnetic, and spin–phonon coupling parameters, we apply FD-FT THz-EPR spectroscopy on two Fe­(II) complexes, both of which are prepared as high-spin complexes but belong to well-known families of Fe­(II) complexes exhibiting spin-crossover (SCO) behavior (cf. Scheme 1).

**Scheme 1:**
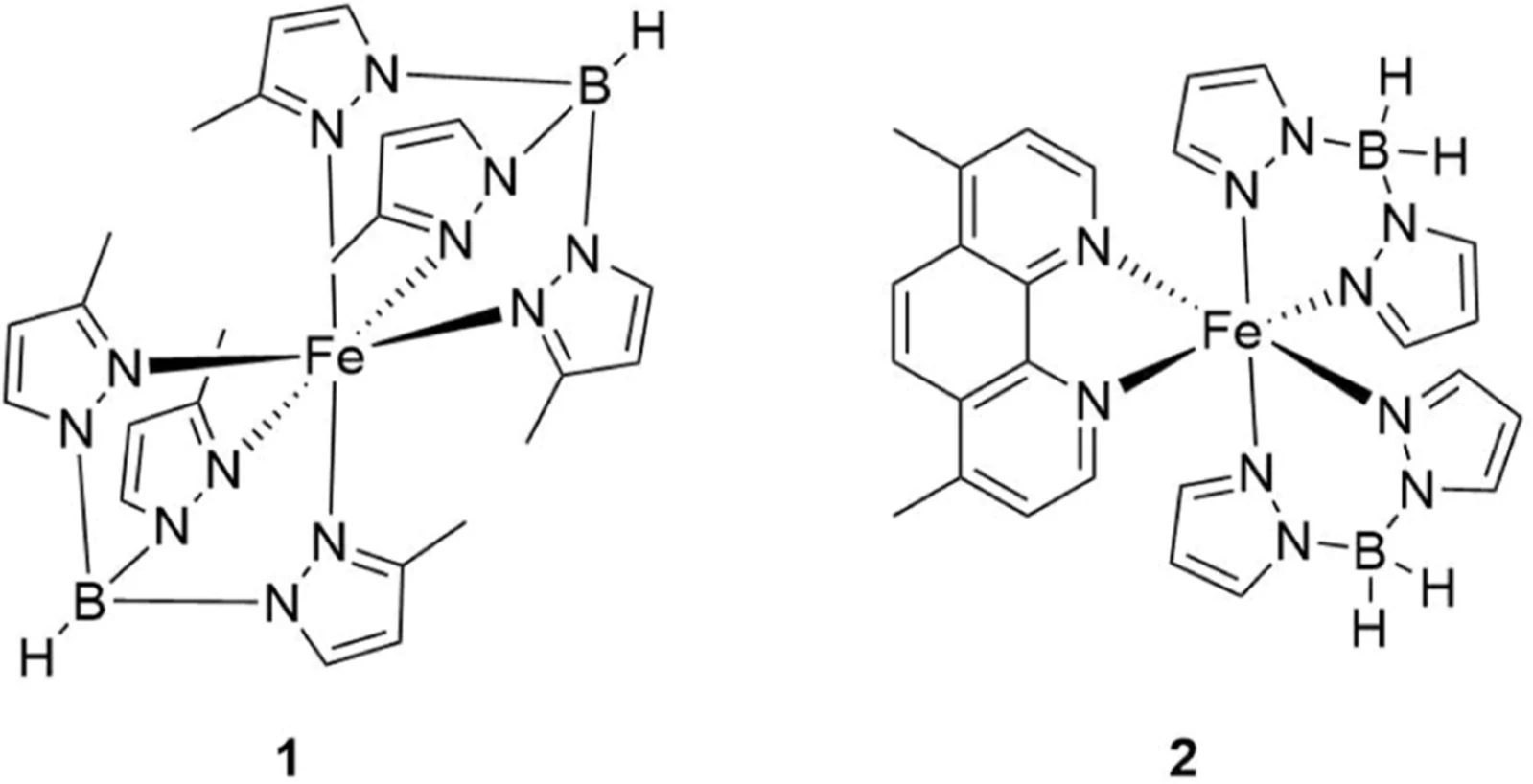
Structure of the HS-Locked Complexes [Fe­(3-Metpz)_2_] (**1**) and [Fe­(bpz)_2_(4,7-DiMephen) (**2**)

The first compound considered in this study, [Fe­(3-Metpz)_2_] (**1**; 3-Metpz = hydrotris­(3-methylpyrazolyl)­borate; cf. Scheme 1), is a member of the well-known family of “scorpionate” complexes.^13^ The synthesis and properties of **1** were first described by Calogero et al. in 1994.^14^ Alternative denominations for **1** are (TpMe, H)_2_Fe (or just (TpMe)_2_Fe). The room-temperature single-crystal structure determination of **1** was found to be consistent with a Fe­(II) high-spin configuration. The Mössbauer spectra of **1** at 4.2 K revealed a magnetically split component (22% of the total intensity) which was attributed to magnetic ordering. The rest of the spectrum contained two doublets assigned to the high-spin (58%) and the low-spin state (20% of the total intensity) of **1**, respectively. Based on these observations, **1** was considered as a spin-crossover complex with properties intermediate between those of the parent (unmethylated) low-spin complex [Fe­(tpz)_2_] (**3**; tpz = hydrotris­(pyrazolyl)­borate) and the dimethylated analogue, [Fe­(3,5-DiMetpz)_2_] (**4**; 3,5-DiMetpz = hydrotris­(3,5-dimethylpyrazolyl)­borate), identified as a high-spin complex by these authors.

Later on, the mentioned data as well as their interpretation were questioned by other groups. With respect to the 3,5-dimethyl-substituted complex **4**, Iasco et al. found evidence for a full thermal spin transition, both in the bulk material and in thin films generated by vacuum sublimation.^15^ Moreover, Mössbauer spectra of complex **1** containing the 3-Metpz ligand were published, which reflect a full spin transition as well, with the 4.2 K spectrum exclusively showing the low-spin state.^16^ In this publication, the differences to the results of Calogero et al. were attributed to the fact that the sample of the former study had been partially oxidized. However, this explained the presence of the magnetically split spectrum as being due to the partial formation of high-spin Fe­(III), it did not account for the difference in the spin-transition behavior of the Fe­(II) fraction of **1** (partial SCO in ref 14, full SCO in ref 16). In particular, the conflicting reports regarding the spin state of **1** at 4.2 K (60% high-spin in ref 14, 100% low-spin in ref 16) called for further study.

On the described background, we decided to reinvestigate compound **1** and found that its physicochemical properties strongly depend upon the applied synthetic method. If it is synthesized according to Calogero et al.^14^ and recrystallized from toluene, it is 100% HS at all temperatures. Notably, other synthetic protocols of **1** (including recrystallization from dichloromethane) generate products exhibiting nonvanishing LS-fractions. This observation was not unexpected as derivatives of **1** with other pyrazole ring substituents have polymorphs exhibiting different magnetic and spin-crossover properties.^16^ It thus appears that the ligand-field strength of the 3-Metpz ligand is very close to the crossover point between HS and LS for Fe­(II) systems and the energies of these states thus very sensitively depend on the crystalline environment in solid samples of [Fe­(3-Metpz)_2_] (**1**). In any case, having pure, well-defined high-spin compound **1** at hand now renders the *S* = 2 state of the prominent class of spin-switchable Fe­(II)-tris­(pyrazolyl)­borate complexes accessible to low-temperature, high-resolution magnetic resonance spectroscopy.

The second complex investigated in this study, [Fe­(bpz)_2_(DiMephen)] (**2**; bpz = dihydrobis­(pyrazolyl)­borate; DiMephen = 4,7-dimethyl-1,10-phenanthroline), was established while studying the effect of vacuum-evaporation on the spin-state-switching properties of the complexes [Fe­(bpz)_2_(phen)], [Fe­(bpz)_2_(bipy)] and derivatives.^17–22^ Notably, **2** shows thermal spin-crossover in vacuum-deposited films (in analogy to parent [Fe­(bpz)_2_(phen)])^23,24^ but remains in HS over the entire temperature range in the crystalline bulk material. Based on X-ray-structural information on related systems, the high-spin state in the bulk is supposedly due to the formation of dimers through π–π interactions between the DiMephen ligands of neighboring complexes.^18,25^ These interactions hinder the contraction of the individual molecules accompanying spin crossover and, if sufficiently strong, fully suppress the thermal spin transition, effectively “locking” the system in the high-spin state. Based on this property, **2** was later on studied with magnetic susceptibility as well as low-temperature saturation magnetization and magnetic Mössbauer spectroscopic measurements.^17^ The fact that its magnetic properties could be described with a Spin-Hamiltonian (SH) indicated that its ^5^T_2_ ground state is split in the *C*_2_ symmetry of the complex with an orbital singlet lying lowest, well separated from higher electronic states. This in turn was important to exclude the assignment of “giant Raman features” observed for **2** and related systems at low temperatures to electronic Raman transitions.^17^

In the following sections, we first present the synthesis as well as the structural and spectroscopic characterization of high-spin locked complex **1**; corresponding data of **2** have been published previously.^18^ Then, the FD-FT THz-EPR spectra of **1** and **2** are presented and analyzed in detail to obtain the respective SH parameters. Moreover, high-level quantum chemical calculations are performed to theoretically determine these SH parameters, showing excellent agreement with the experimental values.

## Results and Discussion

### Preparation of [Fe­(3-Metpz)_2_]

[Fe­(3-Metpz)_2_] (**1**) was prepared by mixing aqueous solutions of FeCl_2_ and [K­(3-Metpz)_2_] in a molar ratio of 1:2 under inert atmosphere and vigorous stirring. The product precipitated immediately as a colorless, finely divided powder and was washed with water and methanol. To obtain microcrystals with high crystal quality, the colorless powder was recrystallized from abs. toluene by dissolution of the powder at room temperature and evaporation through slow stirring under a constant stream of N_2_. Samples of both the powder and the microcrystals were characterized by Mössbauer spectroscopy and XRD. [Fe­(bpz)_2_(DiMephen)] (**2**) was prepared as described previously.^18^

Analytical results of compound **1** show that the powder sample exhibits partial SCO to approximately 80/20% HS/LS upon cooling to 80 K, which is in line with the observation of Calogero et al. (cf. Figure 1, top).^14^ To effectively lock compound **1** in the HS state, the crystallinity of the sample had to be improved, which was achieved by dissolving the powder in toluene and evaporating the solvent slowly by means of a weak but steady stream of dry nitrogen at room temperature. The resulting sample shows almost no residual LS-fraction (2.3%) at 80 K (Figure 1 bottom).

**Figure 1:**
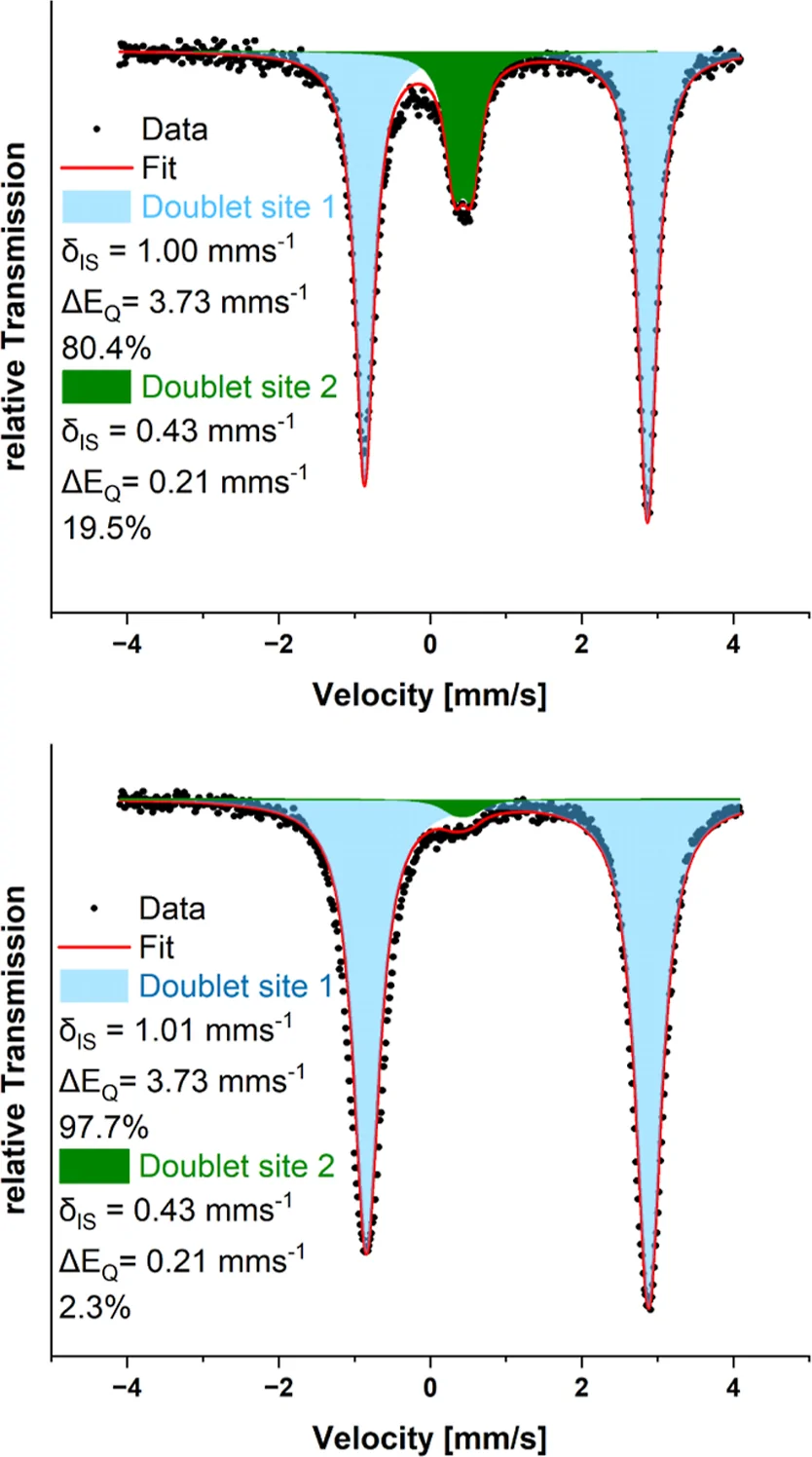
Mössbauer spectra of the powdery sample (top) and the recrystallized sample (bottom) of **1** at 80 K.

The XRD pattern of the powdery sample shows very broad reflexes but no amorphous background, thus implicating that this morphology contains crystallites in the nanoscopic range (cf. Figure 2), while the XRD of the microcrystalline sample shows sharp and well-resolved reflexes. This implies that only complexes in less restricted environments, for example, at crystal edges or near defects, are able to switch to the LS state, while a far-ordered crystal lattice inhibits that capability. This is supported by the finding that better crystal quality, especially larger crystal size, suppresses the SCO almost completely.

**Figure 2:**
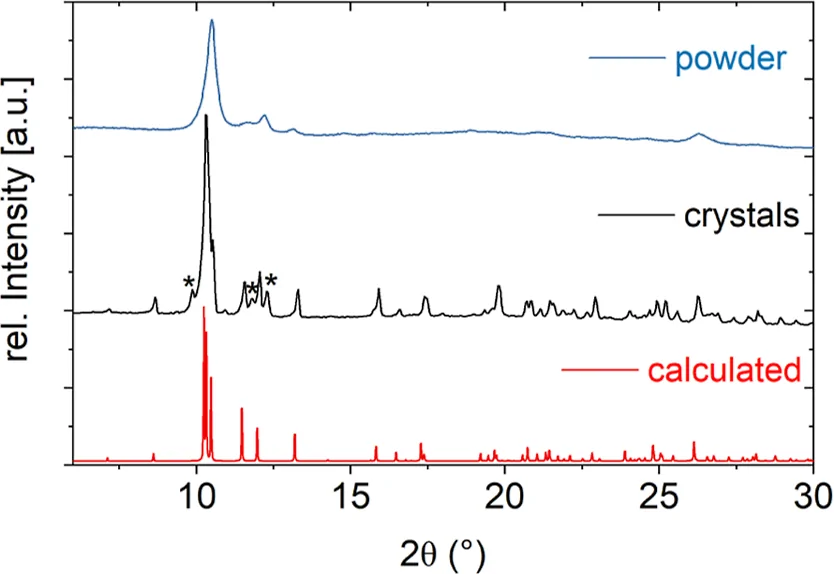
Powder diffraction patterns of **1**. The powdery sample (blue) shows very broad reflexes indicating nanocrystallinity. The recrystallized sample (black) shows sharp reflexes and good resolution, indicating high crystalline quality. Reflexes marked with an asterisk are reflexes of a byphase that is assigned to a crystal phase of **1** containing solvent. As a reference, the curve calculated from crystal structure data is given (red).

As described before by Ossinger et al., compound **2** is locked in the HS state as a bulk sample and shows SCO behavior only when prepared as very thin films.^18,19^ Thus, the two SCO-compounds studied in this work indeed represent examples of HS-locked systems.

### Electronic Structure Calculations

#### [Fe­(3-Metpz)_2_] (**1**)

In order to obtain information on the electronic structure of **1**, NEVPT2-AILFT (N-electron valence state perturbation theory-ab initio ligand field theory) calculations were performed. The results are shown in Figure 3A. We adopt a notation consistent with effective *D*_3*d*_ symmetry of **1**, defining the 3-fold axis as the *z*-axis. The N–Fe–N bond angles within each chelate ring (subtended by nitrogen atoms of the same ligand) are contracted to less than 90°. Consequently, the coordination geometry is best characterized as an elongated triangular antiprism, resulting in the d_*z* ^2^_ orbital lowest in energy within the 3d manifold. The d_*xy*_ and d_*x* ^2^–*y* ^2^_ orbitals are essentially nonbonding and reside slightly higher in energy; under this symmetry notation, these three orbitals correspond to the t_2g_ set of the *O*_*h*_ point group. Conversely, the d_*xz*_ and d_*yz*_ orbitals exhibit antibonding character and thus are highest in energy, corresponding to the e_g_ orbitals in *O*_*h*_ symmetry.

**Figure 3:**
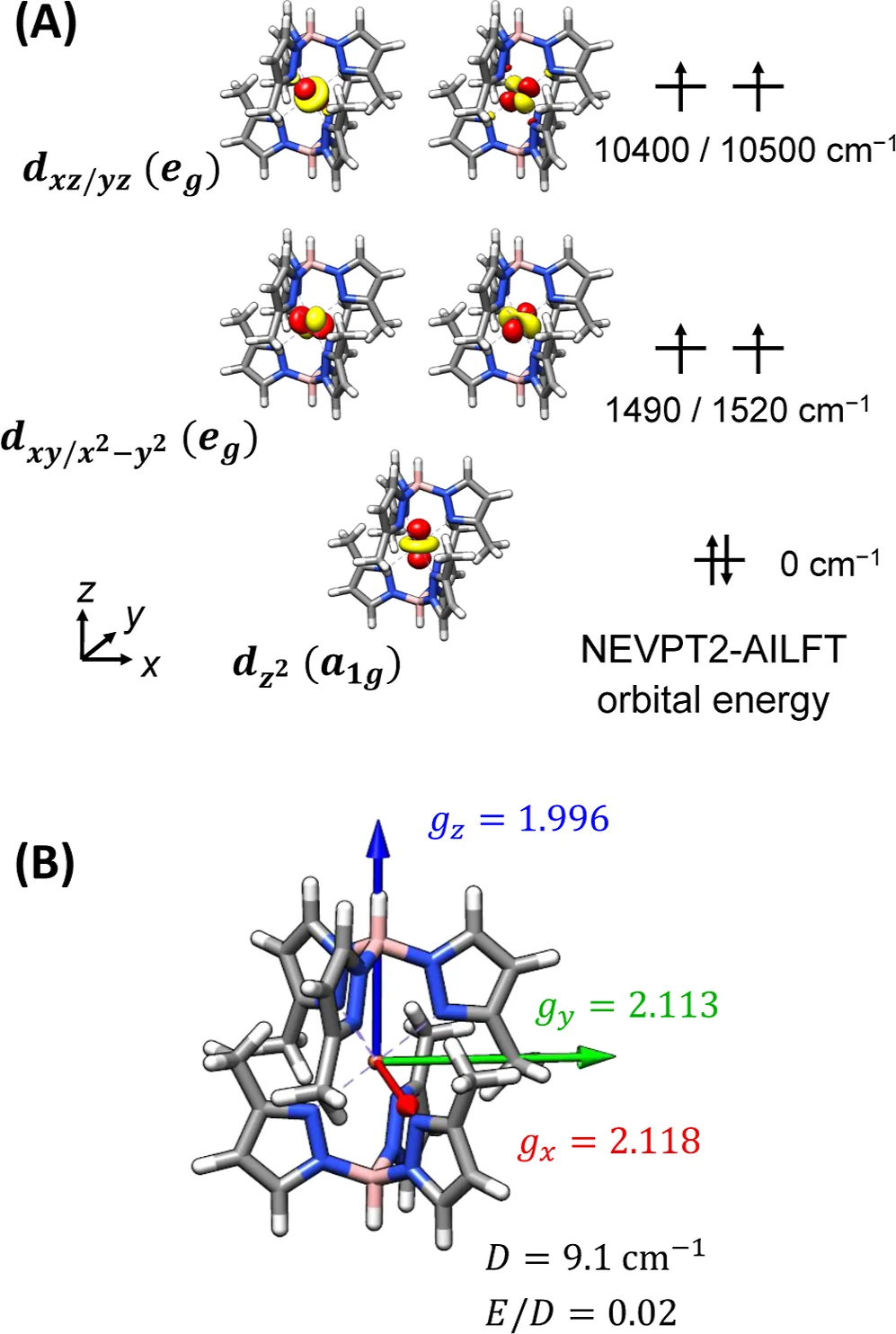
(A) NEVPT2-AILFT results of **1** and (B) calculated SH parameters. The orbital labels are given for effective *D*_3*d*_ point group symmetry.

The corresponding energy level scheme is given in Figure 4A. The two lowest excited states are at 2050 cm^–1^ and 2100 cm^–1^. Two higher levels of *E*_g_ parentage are at 11,100 cm^–1^ and 11,120 cm^–1^ respectively.

**Figure 4:**
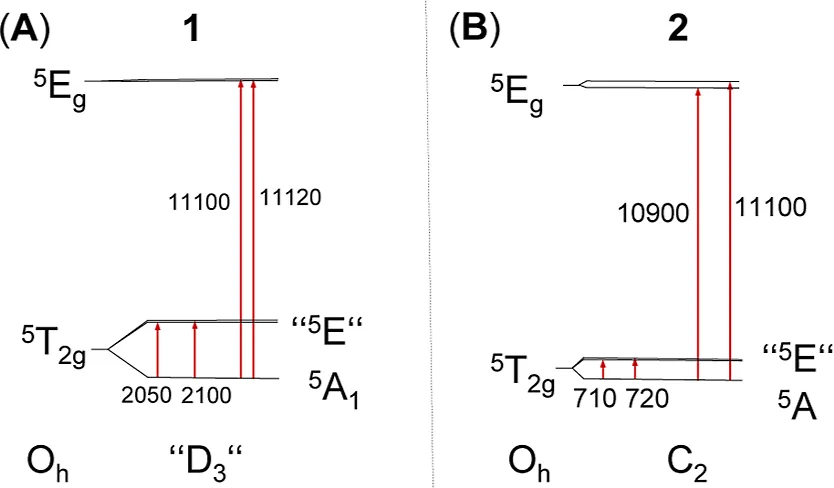
Schematic energy level scheme of **1** (A) and **2** (B) with transition energies in cm^–1^.

HS Fe­(II) (*S* = 2) is a non-Kramers ion with a zero-field splitting (ZFS) of the order of several cm^–1^.^6–10^ For such molecules, if they exhibit an orbitally nondegenerate and well-separated ground state, the spin system can be described by a spin Hamiltonian (SH)

1 $${Ĥ}_{S}=D\left({Ŝ}_{z}^{2}-\frac{1}{3}{Ŝ}^{2}\right)+E\left({Ŝ}_{x}^{2}-{Ŝ}_{y}^{2}\right)+\mu_{B}BgŜ$$

where *D* and *E* are the axial and rhombic ZFS parameters, respectively, and the last term describes the Zeeman interaction including the *g*-tensor. In case of positive *D* (easy-plane anisotropy), the level with *M*_S_ = 0 is lowest in energy. Without an applied magnetic field and neglecting rhombicity (*E* = 0), states of equal |*M*_S_| are degenerate with relative energies of −2*D*, −*D*, and +2*D* (cf. Figure 5). Rhombicity (*E* ≠ 0) induces mixing of states and renders *M*_S_ not to be a good quantum number. Furthermore, the energy levels are shifted relative to the initial values, resulting in lifting of the degeneracy of the initially *M*_S_ = ±1 and *M*_S_ = ±2 levels (cf. Figure 5). Applying a magnetic field, the energy levels split further according to the Zeeman interaction.

**Figure 5:**
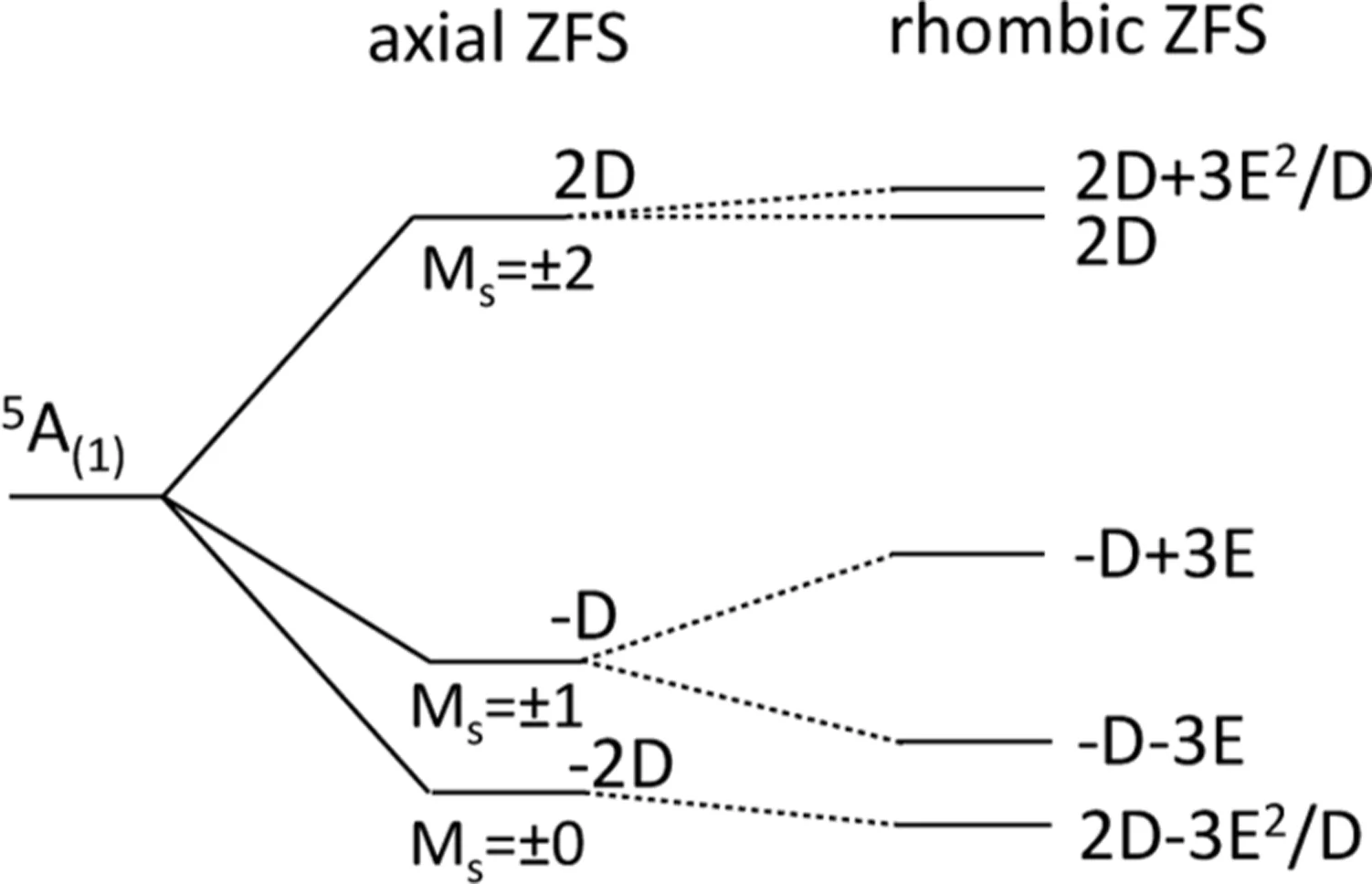
Schematic spin energy level diagram for a ^5^A_(1g)_ state in case of an axial and rhombic ZFS.

In 3d transition-metal systems, the *g*-tensor and ZFS are predominantly governed by spin–orbit coupling (SOC) within the 3d manifold. All excitations within the quintet manifold involve the promotion of an electron from the d_*z* ^2^_ (a_1g_) orbital to the e orbital sets (cf. Figure 3). SOC between the ^5^A_1g_ ground state and the ^5^E excited states (cf. Figure 4) generates unquenched orbital angular momentum along the molecular *x* and *y* axes. This interaction produces a positive ZFS and positive *g*-shifts along the *x* and *y* directions (cf. Figure 3B). The calculation yields SH parameters of *D* = 9.1 cm^–1^, *E*/*D* = 0.02, *g*_*x*_ = 2.118, *g*_*y*_ = 2.113, and *g*_*z*_ = 1.996 (cf. Table 1). These values show excellent agreement with parameters determined from FD-FT THz-EPR spectroscopy (see below). Figure 3B also depicts the orientation of the principal axes of the *g*-tensor in the molecules which are collinear with the principal axes of the ZFS-tensor.

**Table 1:** Best Parameters for the Numerical Simulation Based on Equation 1 of the THz EPR Spectra of **1** and **2** in Figures 7B and 9A, Resepectively, along with the Values Obtained from CASSCF/-NEVPT2 Calculations^a^

parameter	[Fe(3-Metpz)_2_] (**1**)		[Fe(bpz)_2_(DiMephen)] (**2**)	
spin system	experimental	calculated	experimental	calculated
*g*_*x*_	2.2(1)	2.118	2.4(2)	2.297
*g*_*y*_	2.3(1)	2.113	2.45(5)	2.226
*g*_*z*_	2.00(5)	1.996	2.0(5)	1.979
*D*/cm^–1^	10.5(1)	9.1	15.41(5)	17.8
*E*/*D*	0.035(5)	0.02	0.250(2)	0.14

a The simulation for **2** was extended by including spin–phonon coupling (cf. Figure 9B and Table S2).

#### [Fe­(bpz)_2_(DiMephen)] (**2**)

**2** exhibits a lower degree of symmetry relative to **1**; consequently, a distorted octahedral coordination framework is adopted for the description of the electronic structure. The crystal structure reveals six Fe–N bonds with comparable distances of approximately 2.2 Å. Notably, the chelate angle formed by the iron center and the two nitrogen atoms of the phenanthroline ligand is 74.7°, significantly smaller than the ideal 90° in octahedral coordination. This results in the d-orbital with lobes oriented perpendicular to the phenanthroline plane as the lowest within the 3d manifold (Figure 6A).

**Figure 6:**
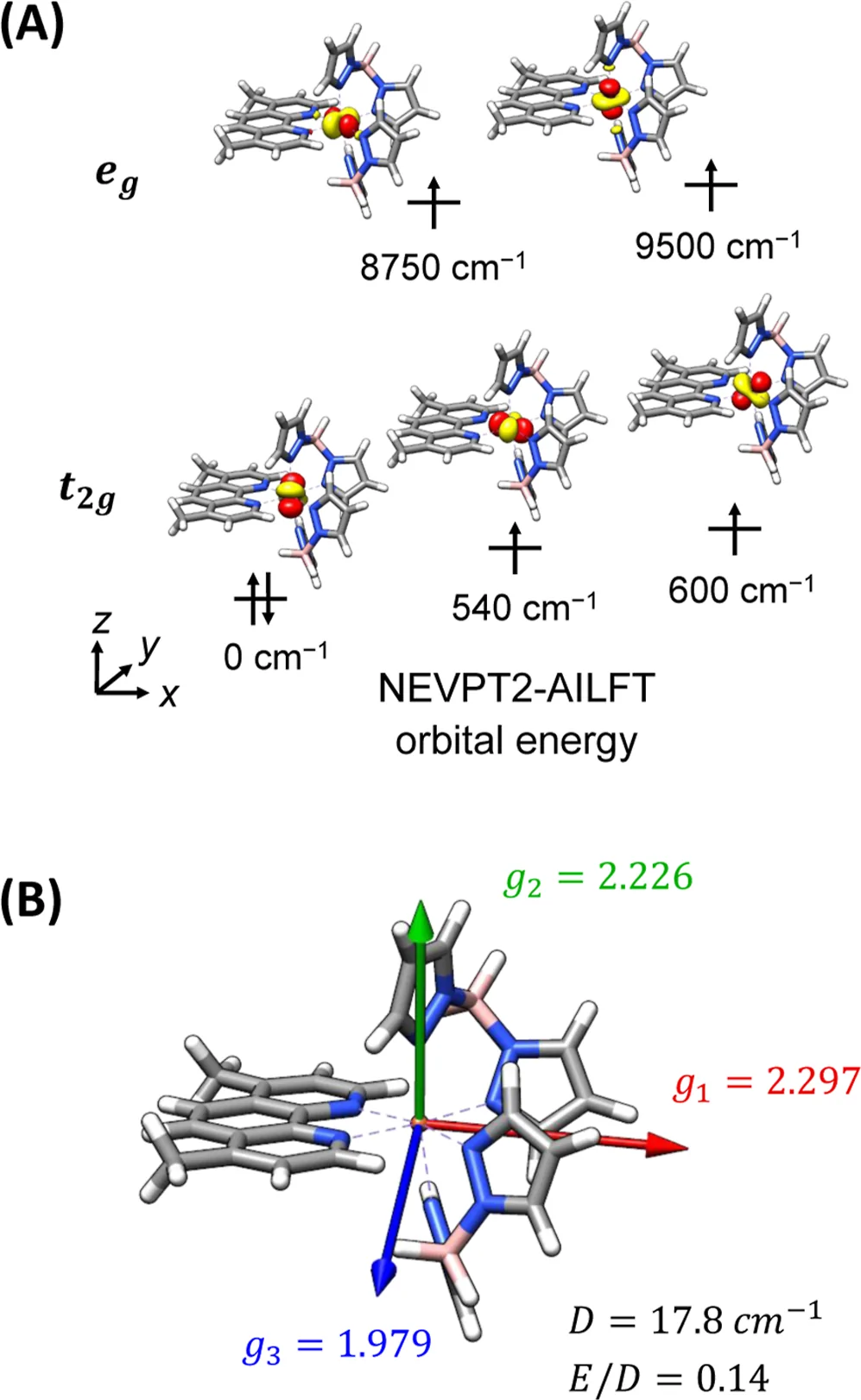
(A) NEVPT2-AILFT results of **2** and (B) calculated SH parameters.

The lowest excited levels of *E* symmetry predicted by CASSCF/-NEVPT2 are at 710 and 720 cm^–1^, the two higher lying levels of *E*-symmetry being at 10,900 cm^–1^ and 11,000 cm^–1^ (cf. Figure 4B). This indicates that the splitting within the t_2g_ orbital set is smaller than in complex **1** (cf. Figure 4A), which leads to an increased contribution of orbital angular momentum, generating a more pronounced *g*-shift and a larger ZFS. This is also likely responsible for the enhanced spin–phonon coupling features identified in the THz EPR spectrum of **2**, which are absent in **1** (see below). Since the second-order mixing induced by SOC is approximately inversely proportional to the excitation energy, the magnetic properties become significantly more sensitive to the vibrational modulation of d-orbital energy levels with lower transition energy.^12^ Calculation yields the SH parameters of *D* = 17.8 cm^–1^, *E*/*D* = 0.14, *g*_*x*_ = 2.297, *g*_*y*_ = 2.226, and *g*_*z*_ = 1.979 (cf. Table 1). The principal axes of the *g*-tensor and *D*-tensor are collinear, yet they are not coaxial with any of the metal–ligand bonds, as illustrated in Figure 6B.

### FD-FT THz-EPR Spectroscopy

#### [Fe­(3-Metpz)_2_]­(**1**)

After experimentally confirming the complete HS nature of compounds **1** and **2** at low temperatures, the electronic structure was further investigated by EPR spectroscopy. Due to the large ZFS, HS Fe­(II) complexes are challenging to study with conventional EPR methods. Therefore, FD-FT THz-EPR in the range of 3–70 cm^–1^ and at magnetic fields up to 10 T was applied to induce transitions between the different magnetic microstates.

The results of the FD-FT THz-EPR measurements on [Fe­(3-Metpz)_2_)] are shown in Figure 7. Experimental data are depicted as magnetic-field division spectra (MDS), obtained by dividing a transmittance spectrum recorded at a particular field *T*( ν̃ ,*B*_0_) by a transmittance spectrum recorded at one field step higher *T*( ν̃ ,*B*_0_ + Δ*B*_0_). The field step Δ*B*_0_ is given in the figure caption of the respective experiment (see also below). This approach effectively cancels out all nonmagnetic contributions. A set of strong signals is detected centered around 11 cm^–1^ at 0 T (**set A**) and splits into several branches upon increasing field, reaching values between <5 and 25 cm^–1^ at 6.5 T. A second set of signals (**set B**) with lower intensity relative to **set A** is centered around 31.5 cm^–1^ at 0 T and splits into two branches shifting to approximately 25 and 37 cm^–1^ at 6.5 T. The THz-EPR spectrum could be simulated based on eq 1 with excellent agreement to the theoretical values yielding the optimal parameters *g*_*x*_ = 2.2, *g*_*y*_ = 2.3, *g*_*z*_ = 2.00, *D* = +10.5 cm^–1^, and a small rhombic distortion of *E*/*D* = 0.035 (cf. Tables 1 and S1).

**Figure 7:**
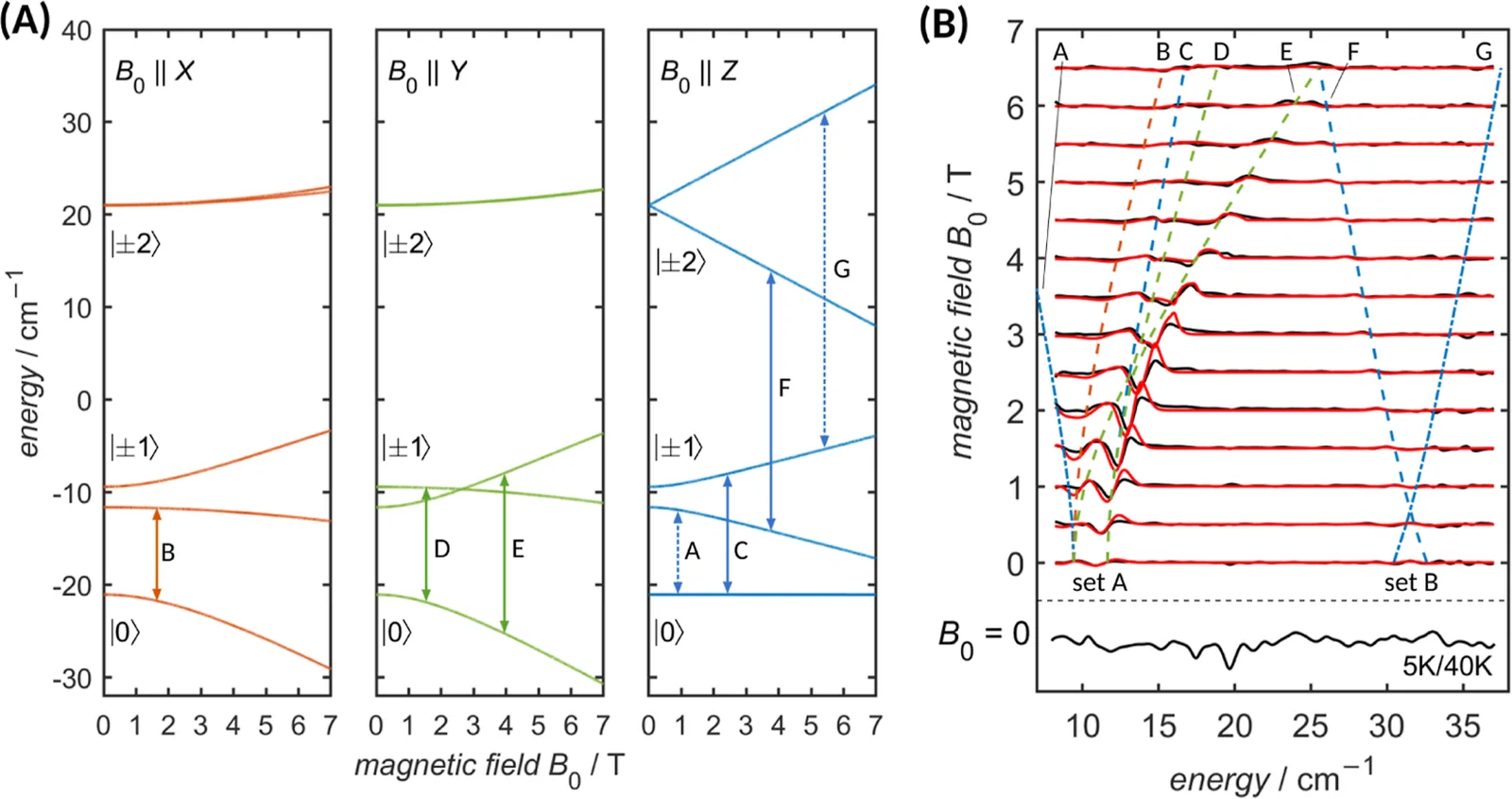
(A) Magnetic-field-dependent spin energy level diagram calculated using the obtained best-fit parameters for the simulations shown in (B). (B) MDS of **1** recorded at a temperature of 5 K (black) and best-fit simulations (red) based on eq 1. The MDS were calculated as relative transmittance by division of a spectrum recorded at the marked magnetic field by a spectrum recorded at 0.5 T higher. The lower trace shows a temperature-division spectrum at zero-field. The assigned EPR transitions labeled A–G are marked as double arrows in (A) for the magnetic field *B*_0_ aligned along *X*- (orange), *Y*- (green), and *Z*- (blue) axes of the ZFS tensor and as dotted lines in (B) using the same color code.

In order to assign the observed transitions, the magnetic-field-dependent spin energy level diagram was calculated by using the obtained SH parameters (Figure 7A). As demonstrated in the diagram, the *M*_S_ = ±1 levels are only slightly split at zero field due to the rhombicity, while the splitting of the *M*_S_ = ±2 is even less, as expected from theory.^26^ Due to the small rhombicity, the nature of the pure *M*_S_ states is mainly retained, and, for the sake of simplicity, the nomenclature will therefore be kept. Thus, **set A** could be assigned to transitions between the ground (*M*_S_ = 0) and the next higher *M*_S_ = ±1 state, while **set B** arises due to transitions involving the *M*_S_ = ±1 and *M*_S_ = ±2 states, which is in line with the expected energy difference of ≈3*D* at 0 T (individual transitions are labeled in Figure 7A). This confirms the positive sign of *D* obtained in the simulation, as for a negative *D*, the signal at ≈3*D* would involve the ground state with a much higher relative intensity as compared to the signal at *D*. Note that in the MDS, the intensity is proportional to both the intensity of the EPR absorption and the magnitude of the field dependence of the transition. Due to the easy-plane magnetization behavior of the (ground) *M*_S_ = 0 level, strongest signals of **set A** in the MDS are observed for orientations of the magnetic field perpendicular to the *z* axis of the ZFS tensor. In contrast, strongest transitions detected in **set B** arise from orientations parallel to the *z*-axis of the ZFS tensor, as, for the *M*_S_ = ±2 levels, these orientations show a pronounced field dependence. Their Zeeman splitting is basically linear over the entire range due to the almost vanishing rhombicity splitting, while perpendicular orientations are barely affected by the external field.

The relative intensity pattern could best be simulated using a temperature of 8(2) K, instead of the experimental set temperature of 5 K (*k*_B_ *T* ≈ 3.5 cm^–1^), shifting the thermal equilibrium population more to the first excited state, which lies at around ≈10.5 cm^–1^ relative to the ground state. This significantly higher temperature may be explained by locally heating of the sample or due to saturation of the lower-energy transitions due to the intense synchrotron THz radiation.

#### [Fe­(bpz)_2_(DiMephen)] (**2**)

The results of the FD-FT THz-EPR measurements on **2** are shown in Figures 8 and 9. Experimental data are again depicted as MDS. SH parameters have been determined before for this system by SQUID magnetometry, with an axial ZFS parameter of about 17 cm^–1^ and a large rhombicity (*E*/*D*) of 0.22,^17^ in good agreement with the calculation (see above and Table 1). Thus, the low-energy part of the expected zero-field THz-EPR spectrum would not be covered with the same setup as used for **1**, which has a spectral window starting only at approximately 8 cm^–1^, which made the use of two different experimental setups necessary (see the Experimental Section for details). A branch of transitions at very low energies around 5 cm^–1^ (**set A**) was detected in the spectral window of 2 cm^–1^ to 22 cm^–1^ (Figure 9A left), while a branch at higher energies (**set B**) was probed in a spectral window of approximately 8 cm^–1^ to 60 cm^–1^ (Figure 9A right). The signals of **set A** shift to slightly higher energies but broaden very quickly, so that at fields above 2 T, the intensity vanished. For **set B**, a signal around 30 cm^–1^ at 0 T was detected, which splits in several branches, shifting to higher energies between 35 and 45 cm^–1^ at 10 T, while the branch shifting to 45 cm^–1^ gains in intensity with increasing fields.

**Figure 8:**
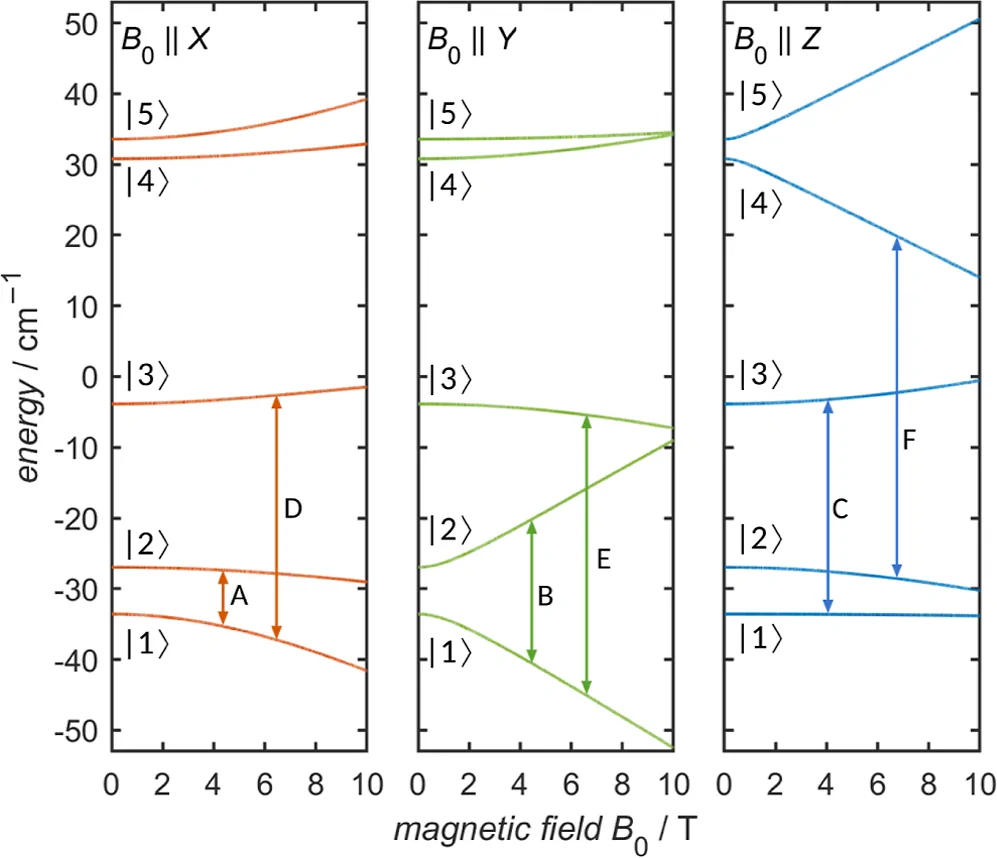
Magnetic-field-dependent spin energy level diagram calculated using the obtained best-fit parameters for the simulations shown in Figure 9(A). The assigned EPR transitions (A–F) are marked as double arrows for the magnetic field *B*_0_ aligned along *X*- (orange), *Y*- (green), and *Z*- (blue) axes of the ZFS tensor.

**Figure 9:**
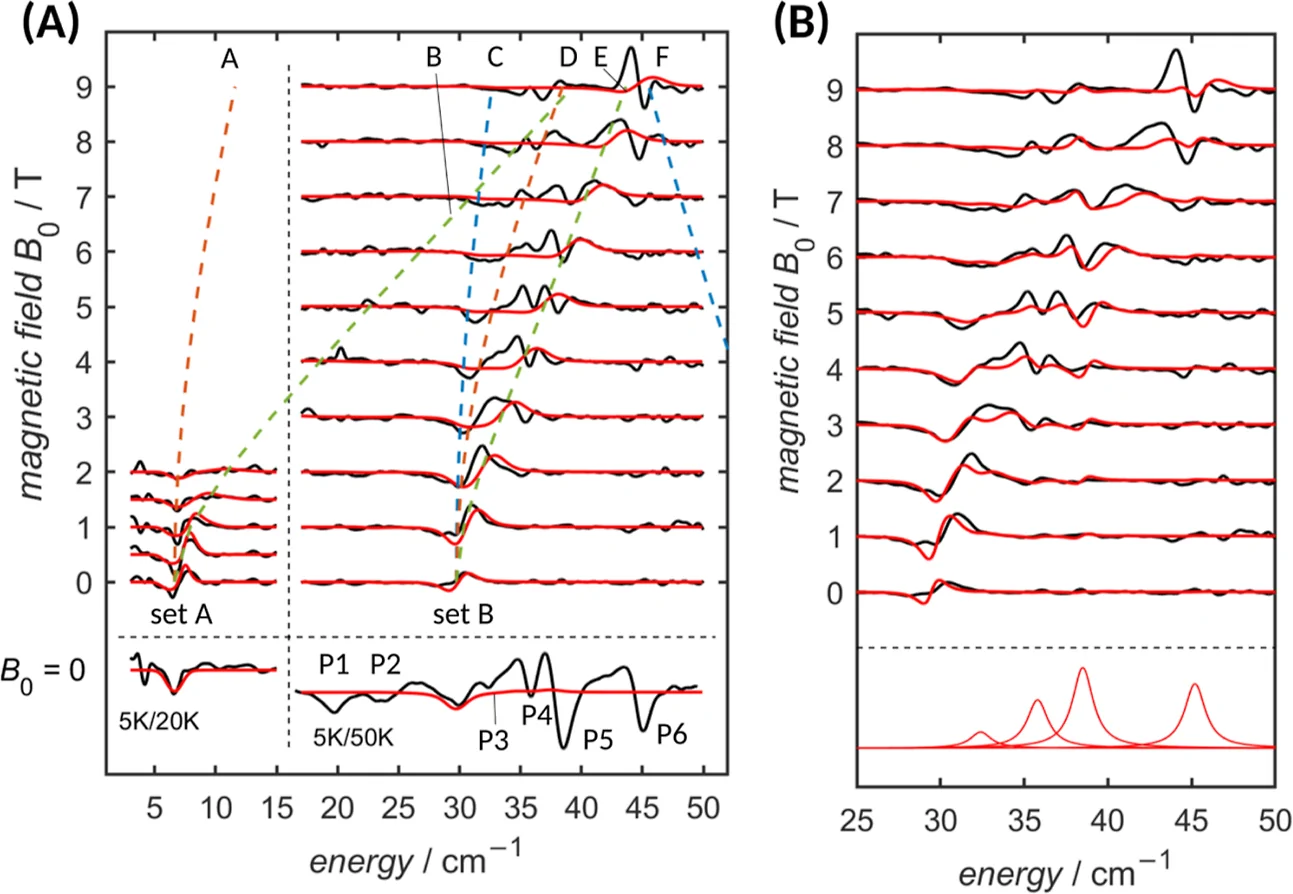
(A) Lower traces: temperature-division spectra (TDS) of **2**, recorded at zero field. Upper traces: MDS of **2**; the MDS were calculated as relative transmittance by division of a spectrum recorded at the marked magnetic field by a spectrum recorded at 1 T higher. The assigned EPR transitions (A–F) are marked as dotted lines using the same color code as in Figure 8. Assigned phonons in the TDS are labeled as P1–P6. (B) Fit including spin–phonon coupling (red). The lower trace shows the phonons included in the simulation.

The lower traces in Figure 9A show temperature-dependent experiments at zero-field represented as temperature-division spectrum (TDS). Here, additional signals were detected, around 6.5 cm^–1^ in the low-energy range and around 19.8, 23.7, 29.9, 32.3, 35.8, 38.5, and 45.2 cm^–1^ in the high-energy range. As in the TDS nonmagnetic contributions are not canceled out, both, magnetic signals and phonons, are detected. Extrapolating the propagation of the field-dependence of **sets A** and **B** in the MDS to 0 T, the two magnetic signals in the TDS could, however, clearly be identified as the bands at 6.5 and 29.9 cm^–1^, which present the zero-field EPR transitions. Comparing this with the theoretically predicted/experimentally determined ZFS parameters,^17^ these can be attributed to transitions from the ground state to the two lowest lying excited states, which are separated by 6*E* ≈ 23 cm^–1^. The other peaks in the TDS are assigned to phonons and referred to as P1–P6 with rising transition energy (see Figure 9A).

With the zero-field transitions at hand, accurate values for *D* and *E* are available which can serve as starting values for spectral simulations. However, in contrast to the MDS of complex **1**, where the positions and intensities of EPR transitions evolve continuously with increasing the magnetic field (cf. Figure S1), the MDS of complex **2** exhibits numerous interference structures and avoided-crossing features at the positions of phonons P3–P6, as illustrated in the contour plots of the MDS of **2** in Figure 10. These observations provide clear evidence for significant spin–phonon coupling, indicating that the SH described by eq 1 is insufficient to accurately reproduce the THz EPR spectra of **2**. Prior to incorporating spin–phonon coupling effects, the simulation of the THz EPR spectra of **2** was first conducted to obtain the SH parameters in eq 1. Owing to the presence of avoided-crossing features, the simulation was initially optimized using transitions that are unaffected or only weakly influenced by spin–phonon coupling, namely the signals belonging to **set A** and the low-field signals of **set B**. At higher fields, the center of the simulated peak was aligned at the center of the avoided crossing, which is expected to be the transition energy of the EPR resonance in the absence of spin–phonon coupling.^27^ By this approach, the optimal parameters obtained were *g*_*x*_ = 2.4, *g*_*y*_ = 2.45, *g*_*z*_ = 2.0, *D* = +15.41 cm^–1^, and *E*/*D* = 0.250 (cf. Table 1).

**Figure 10:**
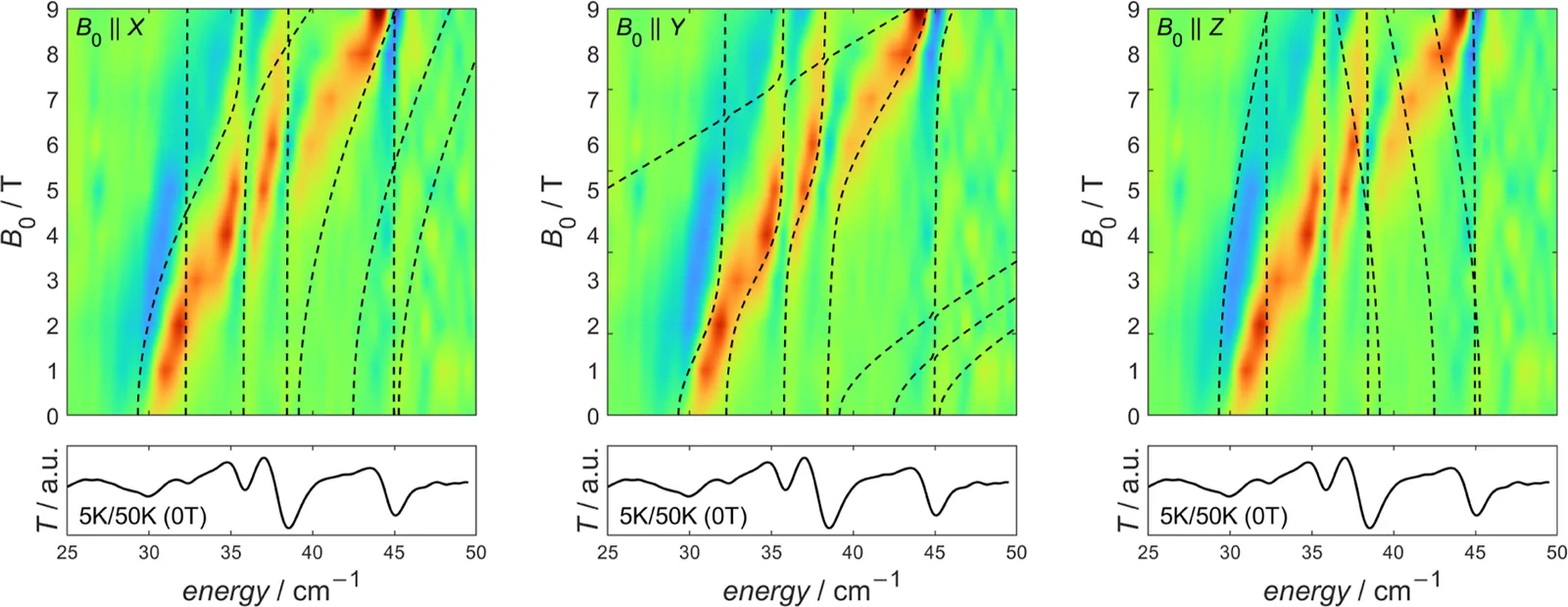
Contour plots (top) of the experimental MDS of compound **2** (cf. Figure 9 upper traces) with respective phonon spectra represented as TDS (bottom, *T*: relative transmittance). Simulated field-dependent transition energies based on the spin–phonon coupling extended SH (eq 2) are superimposed for the magnetic field along *X*-, *Y*-, and *Z*-axes of the ZFS principal axes system.

Based on the results of the simulation, a magnetic-field-dependent spin energy level diagram was calculated to assign the signals to specific transitions (Figure 8). Note that due to the substantial rhombicity, *M*_S_ is not a good quantum number (see above), and in this case, the levels are labeled |1⟩–|5⟩ in energetically rising order. Basically, all detected transitions involve the ground |1⟩ and the first two excited states |2⟩ and |3⟩, respectively. This is attributed to the population of mainly |1⟩ at the experimental temperature of 5 K, although due to extensive mixing of states, transitions involving higher states should have a finite probability and can in principle be observed at higher temperature. Here, transition F is the only detected transition which includes higher excited states, shifting into the spectral window at fields >7 T. Like **1**, compound **2** features easy plane anisotropy; however, due to the high rhombicity, the states |2⟩ and |3⟩ have partial character of the ground state and thus show rather small field dependency. Due to this fact, observed transitions with high intensity mainly arise from nonparallel orientations, especially at low fields. Notably, transition B, which arises from an orientation *B*_0_∥*Y*, quickly loses intensity at fields above 1 T.

Recently, a simulation protocol has been developed which is based on an extended SH that includes the first-order spin–phonon coupling^12^

2 $${Ĥ}_{tot}={Ĥ}_{S}+{Ĥ}_{Ph}+{Ĥ}_{S-Ph}$$

with

$${Ĥ}_{Ph}=\sum_{\alpha }^{N}\hbar \omega_{\alpha }\left({\mathrm{n̂}}_{\alpha }+\frac{1}{2}\right)$$

$${Ĥ}_{S-Ph}=\sum_{\alpha }^{N}\frac{\partial {Ĥ}_{S}}{\partial Q_{\alpha }}{\mathrm{Q̂}}_{\alpha }$$

Here, Ĥ _S_ is the SH in eq 1, Ĥ _Ph_ is an operator describing the energies of the phonons, and Ĥ _S–Ph_ is a spin–phonon interaction Hamiltonian. The summation runs over all modes α. This protocol was subsequently employed to simulate the MDS of complex **2**. The best simulation obtained using this model, which includes phonons P3, P4, P5, and P6, is shown in Figure 9B as red traces, while the corresponding transition energies superimposed on the experimental MDS are presented in Figures 10 and S2. The energies and relative intensities of the uncoupled phonons with respect to the magnetic transitions were determined directly from the raw THz absorbance spectrum recorded at 5 K (Figure S3). The simulation is based on the parameters summarized in Table S2 in the Supporting Information. Owing to the inclusion of four phonons and the presence of overlapping interference features arising from the phonons’ close energetic proximity, the simulation is inherently overparameterized. Consequently, the resulting parameter set cannot be regarded as quantitatively definitive. Nevertheless, the fit now reproduces much better the experimental spectra in the region where heavy spin–phonon coupling occurs; i.e., at fields around 6 T (cf. Figure 9A,B). The present simulations thus provide an estimate of the order of magnitude of the spin–phonon coupling constants and semi-quantitatively reproduce the principal features of the experimental THz EPR spectra.

## Conclusion

Two SCO systems, **1** and **2**, were prepared in a way that retains the high-spin state at low temperatures. This allowed low-temperature FD-FT THz-EPR investigations of these compounds and resulted in the determination of magnetic transitions and avoided crossings due to spin–phonon coupling in compound **2** as well as the respective SH parameters. Theoretical calculations successfully resolved the electronic structure of the complexes, and the SH parameters obtained by spectroscopic analysis were reproduced by in silico methods. The spin–phonon coupling approach, established by Chen et al.,^12^ delivered a remarkable improvement of the simulated spectra of **2** and produced fits with high accordance to the measured data. This is due to a combination of the spectroscopic and theoretical methods. Moving forward, the foundation for the interpretation of THz-EPR spectra is laid, especially regarding spin–phonon coupling phenomena. Although there is still room for improvement in the exact consideration of phonon positions and intensities, the results can be regarded as a substantial improvement in the spectroscopic characterization of the Fe­(II) SCO compounds.

## Experimental Section

### Frequency-Domain Fourier-Transform Electron Paramagnetic Resonance

Frequency-domain Fourier-transform THz-EPR, or FD-FT THz-EPR in short, was performed at the THz beamline at the electron storage ring BESSY II at the Helmholtz-Zentrum Berlin (HZB). The experimental principle is described in detail elsewhere.^3^ Coherent synchrotron radiation provided through the low-α operation mode of BESSY II^28^ was used to cover the THz- and sub-THz-range bands to detect the magnetic transitions and to cope with strong nonmagnetic absorbance of the powder material (40.4 mg ([Fe­(3-Metpz)_2_]) and 40.3 mg [Fe­(bpz)_2_(DiMephen)] as pressed into 100 mg of polyethylene (PE) pellets. All measurements shown here were performed using a 12 T magnet (Croygenic Ltd., UK) equipped with a 2–320 K variable temperature insert in Voigt geometry (radiation propagation direction k⃗ perpendicular to the external magnetic field ${\mathrm{B⃗}}_{0}$ ). Using a three-mirror polarization retarder, the THz magnetic field ${\mathrm{B⃗}}_{1}$ can be set to be parallel, perpendicular or inclined at any angle to ${\mathrm{B⃗}}_{0}$ . As a detector, either a liquid He-cooled 4.2 K and/or a 1.6 K Si bolometer (Infrared Laboratories) was used in combination with a suitable beamsplitter (6 μm multilayer, 23 μm or 125 μm Mylar, respectively) inside a BRUKER IFS125 HR FTIR spectrometer. The spectral resolution was selected as 0.5 cm^–1^ (maximum spectral resolution is 0.0063 cm^–1^). Only for recording the high-energy part of the spectrum of compound **2**, unpolarized light emitted from a mercury-arc lamp was used instead of coherent synchrotron radiation, while other parameters stayed the same.

As described in ref 3, nonmagnetic absorption features (e.g., by phonons) are eliminated by obtaining differential magnetic maps consisting of relative transmittance spectra which are divisions of sweeps at a particular field by one field step higher $MDS=\frac{T\left(\mathrm{\nu ̃},B_{0}\right)}{T(\mathrm{\nu ̃},B_{0}+\Delta B_{0})}$ (“magnetic-field division spectra”, MDS) at a given temperature, here at 5 K. Usually reference spectra at 20–50 K are used to determine relative absorbances between Boltzmann-populated and unpopulated states.

Spectral simulations were performed using the Easy Spin Toolbox^29,30^ for MATLAB.

### Computational Setup

All calculations were performed with the ORCA program package (version 6.1).^31^ Single-crystal structures of complexes **1** and **2** are available in refs 14 and 18 ; all hydrogen atoms were optimized with the TPSSh density functional with D3BJ corrections and def2-SVP basis sets.^32–35^ The RIJCOSX approximation was used with the default auxiliary basis set.^36–40^ Single-point calculations were performed using CASSCF combined with N-electron valence state perturbation theory (NEVPT2) with a CAS­(6,5) active space (five 3d orbitals, six electrons).^41–43^ The def2-TZVP basis set was used for all atoms. Five quintets, 45 triplets, and 50 singlets were included in the stage-average calculation, followed by quasi-degenerate perturbation theory to include the SOC. Ab-initio ligand field theory was employed to analyze the electronic structure.^44,45^

## Supplementary Material



## Notes

Contour plots of the MDS of compounds **1** and **2**, detailed information about the determined SH, and spin–phonon coupling parameters as well as the THz absorbance spectrum of compound **2** (PDF)

## References

[1] Gütlich P. (2013). Spin Crossover–Quo
Vadis?. Eur. J. Inorg. Chem..

[2] Gütlich P., Hauser A., Spiering H. (1994). Thermal and Optical Switching of
Iron­(II) Complexes. Angew. Chem., Int. Ed..

[3] Nehrkorn J., Holldack K., Bittl R., Schnegg A. (2017). Recent progress
in
synchrotron-based frequency-domain Fourier-transform THz-EPR. J. Magn. Reson..

[4] Kragskow J. G. C., Marbey J., Buch C. D., Nehrkorn J., Ozerov M., Piligkos S., Hill S., Chilton N. F. (2022). Analysis of vibronic
coupling in a 4f molecular magnet with FIRMS. Nat. Commun..

[5] Walter M., Kuhlemann E. F., Al Said T., Trommer C. W., Tuczek F., Holldack K., Kuch W., Thakur S. (2025). Tracking the HS-HS,
HS-LS, and LS-LS States in the Spin Transition of a Dinuclear Fe­(II)
Complex by Broadband FTIR Spectroscopy. J. Phys.
Chem. Lett..

[6] Champion P. M., Sievers A. J. (1980). Far Infrared Magnetic
Resonance of Deoxyhemoglobin
and Deoxymyoglobin. J. Chem. Phys..

[7] Carver G., Tregenna-Piggott P. L. W., Barra A.-L., Neels A., Stride J. A. (2003). Spectroscopic and Structural Characterization of the
[Fe­(imidazole)_6_]^2+^ Cation. Inorg. Chem..

[8] Telser J., van Slageren J., Vongtragool S., Dressel M., Reiff W. M., Zvyagin S. A., Ozarowski A., Krzystek J. (2005). High-frequency/high-field
EPR spectroscopy of the high-spin ferrous ion in hexaaqua complexes. Magn. Reson. Chem..

[9] Harman W. H., Harris T. D., Freedman D. E., Fong H., Chang A., Rinehart J. D., Ozarowski A., Sougrati M. T., Grandjean F., Long G. J., Long J. R., Chang C. J. (2010). Slow Magnetic Relaxation
in a Family of Trigonal Pyramidal Iron­(II) Pyrrolide Complexes. J. Am. Chem. Soc..

[10] SantaLucia D. J., Devkota L., Lindeman S. V., Ozarowski A., Krzystek J., Ozerov M., Greer S. M., Cummins D. C., Theopold K. H., Atanasov M., Telser J., Fiedler A. T. (2025). Magnetospectroscopic
Studies of a Series of Fe­(II) Scorpionate Complexes: Assessing the
Relationship between Halide Identity and Zero-Field Splitting. Inorg. Chem..

[11] Albino A., Benci S., Atzori M., Chelazzi L., Ciattini S., Taschin A., Bartolini P., Lunghi A., Righini R., Torre R., Totti F., Sessoli R. (2021). Temperature Dependence
of Spin–Phonon Coupling in [VO­(acac)_2_]: A Computational
and Spectroscopic Study. J. Phys. Chem. C.

[12] Chen H., van Gastel M., Schnegg A., Neese F. (2026). Bridging Vibrations
and Spins: Mode-Resolved Spin-Phonon Coupling Revealed through THz
EPR/Magnetic IR Simulation. J. Phys. Chem. A.

[13] Pettinari C. (2016). Scorpionate
Compounds. Eur. J. Inorg. Chem..

[14] Calogero S., Lobbia G., Cecchi P., Valle G., Friedl J. (1994). A Mössbauer
study of some iron­(II) and iron­(III) poly­(pyrazolyl)­borates. The x-ray
crystal structures of iron­(II)­bis­[hydridotris­(3-methyl-1H-pyrazol-1-yl)­borate]
and iron­(II)­bis­[hydridotris­(1H-pyrazol-1-yl)­borate] tetrafluoroborate. Polyhedron.

[15] Iasco O., Boillot M.-L., Bellec A., Guillot R., Rivière E., Mazerat S., Nowak S., Morineau D., Brosseau A., Miserque F., Repain V., Mallah T. (2017). The disentangling of
hysteretic spin transition, polymorphism and metastability in bistable
thin films formed by sublimation of bis­(scorpionate) Fe­(II) molecules. J. Mater. Chem. C.

[16] Reger D. L., Gardinier J. R., Elgin J. D., Smith M. D., Hautot D., Long G. J., Grandjean F. (2006). Structure-Function Correlations in
Iron­(II) Tris­(pyrazolyl)­borate Spin-State Crossover Complexes. Inorg. Chem..

[17] Ossinger S., Naggert H., Bill E., Näther C., Tuczek F. (2019). Electronic Structure, Vibrational
Spectra, and Spin
Crossover Properties of Vacuum Evaporable Iron­(II) Bis­(dihydrobis­(pyrazolyl)­borate)
Complexes with Diimine Coligands. Origin of Giant Raman Features. Inorg. Chem..

[18] Ossinger S., Kipgen L., Naggert H., Bernien M., Britton A. J., Nickel F., Arruda L. M., Kumberg I., Engesser T. A., Golias E., Näther C., Tuczek F., Kuch W. (2020). Effect of
ligand methylation on the spin switching properties of surface supported
spin crossover molecules. J. Phys. Condens.
Matter.

[19] Kipgen L., Bernien M., Ossinger S., Nickel F., Britton A. J., Arruda L. M., Naggert H., Luo C., Lotze C., Ryll H., Radu F., Schierle E., Weschke E., Tuczek F., Kuch W. (2018). Evolution of cooperativity
in the
spin transition of an iron­(II) complex on a graphite surface. Nat. Commun..

[20] Naggert H., Bannwarth A., Chemnitz S., von Hofe T., Quandt E., Tuczek F. (2011). First observation
of light-induced spin change in vacuum
deposited thin films of iron spin crossover complexes. Dalton Trans..

[21] Bernien M., Naggert H., Arruda L. M., Kipgen L., Nickel F., Miguel J., Hermanns C. F., Krüger A., Krüger D., Schierle E., Weschke E., Tuczek F., Kuch W. (2015). Highly Efficient Thermal and Light-Induced
Spin-State Switching of
an Fe­(II) Complex in Direct Contact with a Solid Surface. ACS Nano.

[22] Gopakumar T. G., Matino F., Naggert H., Bannwarth A., Tuczek F., Berndt R. (2012). Electron-Induced Spin
Crossover of
Single Molecules in a Bilayer on Gold. Angew.
Chem., Int. Ed..

[23] Moliner N., Salmon L., Capes L., Muñoz M. C., Létard J.-F., Bousseksou A., Tuchagues J.-P., McGarvey J. J., Dennis A. C., Castro M., Burriel R., Real J. A. (2002). Thermal and Optical Switching of
Molecular Spin States
in the {[FeL­[H2B­(pz)­2]­2} Spin-Crossover System (L = bpy, phen). J. Phys. Chem. B.

[24] Real J. A., Muñoz M. C., Faus J., Solans X. (1997). Spin Crossover in Novel
Dihydrobis­(1-pyrazolyl)­borate [H2B­(pz)­2]-Containing Iron­(II) Complexes.
Synthesis, X-ray Structure, and Magnetic Properties of [FeL­{H2B­(pz)­2}­2]
(L = 1,10-Phenanthroline and 2,2‘-Bipyridine). Inorg. Chem..

[25] Naggert H., Rudnik J., Kipgen L., Bernien M., Nickel F., Arruda L. M., Kuch W., Näther C., Tuczek F. (2015). Vacuum-evaporable spin-crossover
complexes: physicochemical
properties in the crystalline bulk and in thin films deposited from
the gas phase. J. Mater. Chem. C.

[26] Atanasov M., Aravena D., Suturina E., Bill E., Maganas D., Neese F. (2015). First principles approach to the electronic structure, magnetic anisotropy
and spin relaxation in mononuclear 3d-transition metal single molecule
magnets. Coord. Chem. Rev..

[27] Moseley D. H., Stavretis S. E., Thirunavukkuarasu K., Ozerov M., Cheng Y., Daemen L. L., Ludwig J., Lu Z., Smirnov D., Brown C. M. (2018). Spin-phonon couplings
in transition metal complexes
with slow magnetic relaxation. Nat. Commun..

[28] Abo-Bakr M., Feikes J., Holldack K., Kuske P., Peatman W. B., Schade U., Wüstefeld G., Hübers H.-W. (2003). Brilliant,
Coherent Far-Infrared (THz) Synchrotron Radiation. Phys. Rev. Lett..

[29] Stoll S., Schweiger A. (2006). EasySpin,
a Comprehensive Software Package for Spectral
Simulation and Analysis in EPR. J. Magn. Reson..

[30] Nehrkorn J., Schnegg A., Holldack K., Stoll S. (2015). General Magnetic Transition
Dipole Moments for Electron Paramagnetic Resonance. Phys. Rev. Lett..

[31] Neese F. (2025). Software Update:
The ORCA Program System—Version 6.0. Wiley Interdiscip. Rev.: Comput. Mol. Sci..

[32] Staroverov V. N., Scuseria G. E., Tao J., Perdew J. P. (2003). Comparative assessment
of a new nonempirical density functional: Molecules and hydrogen-bonded
complexes. J. Chem. Phys..

[33] Grimme S., Ehrlich S., Goerigk L. (2011). Effect of the damping function in
dispersion corrected density functional theory. J. Comput. Chem..

[34] Grimme S., Antony J., Ehrlich S., Krieg H. (2010). A consistent and accurate
ab initio parametrization of density functional dispersion correction
(DFT-D) for the 94 elements H-Pu. J. Chem. Phys..

[35] Weigend F., Ahlrichs R. (2005). Balanced basis sets of split valence, triple zeta valence
and quadruple zeta valence quality for H to Rn: Design and assessment
of accuracy. Phys. Chem. Chem. Phys..

[36] Izsák R., Neese F., Klopper W. (2013). Robust fitting techniques in the
chain of spheres approximation to the Fock exchange: The role of the
complementary space. J. Chem. Phys..

[37] Izsák R., Neese F. (2011). An overlap fitted chain of spheres exchange method. J. Chem. Phys..

[38] Neese F., Wennmohs F., Hansen A., Becker U. (2009). Efficient, approximate
and parallel Hartree-Fock and hybrid DFT calculations. A ‘chain-of-spheres’
algorithm for the Hartree-Fock exchange. Chem.
Phys..

[39] Kossmann S., Neese F. (2009). Comparison of two efficient
approximate Hartee-Fock approaches. Chem. Phys.
Lett..

[40] Stoychev G. L., Auer A. A., Neese F. (2017). Automatic
Generation of Auxiliary
Basis Sets. J. Chem. Theory Comput..

[41] Malmqvist P.-Å., Roos B. O. (1989). The CASSCF state interaction method. Chem. Phys. Lett..

[42] Angeli C., Cimiraglia R., Malrieu J.-P. (2002). n-electron valence state perturbation
theory: A spinless formulation and an efficient implementation of
the strongly contracted and of the partially contracted variants. J. Chem. Phys..

[43] Angeli C., Cimiraglia R., Evangelisti S., Leininger T., Malrieu J.-P. (2001). Introduction of
n-electron valence states for multireference
perturbation theory. J. Chem. Phys..

[44] Atanasov, M. ; Ganyushin, D. ; Sivalingam, K. ; Neese, F. In Molecular Electronic Structures of Transition Metal Complexes II; Mingos, D. M. P. , Day, P. , Dahl, J. P. , Eds.; Springer Berlin Heidelberg: Berlin, Heidelberg, 2012; pp 149–220.

[45] Lang L., Atanasov M., Neese F. (2020). Improvement
of Ab Initio Ligand Field
Theory by Means of Multistate Perturbation Theory. J. Phys. Chem. A.

