# Supplementary material for: High-Spin-Locked Fe(II) Spin-Crossover Compounds: THz-EPR Investigations and Spin–Phonon Coupling
Source: Inorg Chem. 2026 Oct 14;65(42):24970–9. doi: 10.1021/acs.inorgchem.6c04101 (PMC13715575; doi:10.1021/acs.inorgchem.6c04101)
Supplement: Supplementary file 1 [file ic6c04101_si_001.pdf]

Supporting Information

High-Spin-Locked Fe(II) Spin-Crossover Compounds: THz-EPR Investigations and Spin-Phonon Coupling

Tarek Al Said,[†] Eike Kuhlemann,[‡] Haowei Chen,[¶] Maurice van Gastel,[¶] Marcel Walter,[§] Malte Laurin Schwank,[§] Sangeeta Thakur,[§] Karsten Holldack,[†] Wolfgang Kuch,[*,§] Felix Tuczek,[*,‡] and Frank Neese[*,¶]

†*Helmholtz-Zentrum Berlin für Materialien und Energie GmbH, Hahn-Meitner-Platz 1, 14109, Berlin, Germany*
‡*Christian-Albrechts Universität zu Kiel, Institut für Anorganische Chemie, Max-Eyth-Strasse 2, 24118 Kiel*
¶*Max-Planck-Institut für Kohlenforschung, Department of Molecular Theory and Spectroscopy, Kaiser-Wilhelm-Platz 1, 45470 Mülheim an der Ruhr, Germany*
§*Freie Universität Berlin, Institut für Experimentalphysik, Arnimallee 14, 14195 Berlin-Dahlem, Germany*

E-mail: kuch@physik.fu-berlin.de; ftuczek@ac.uni-kiel.de; neese@kofo.mpg.de

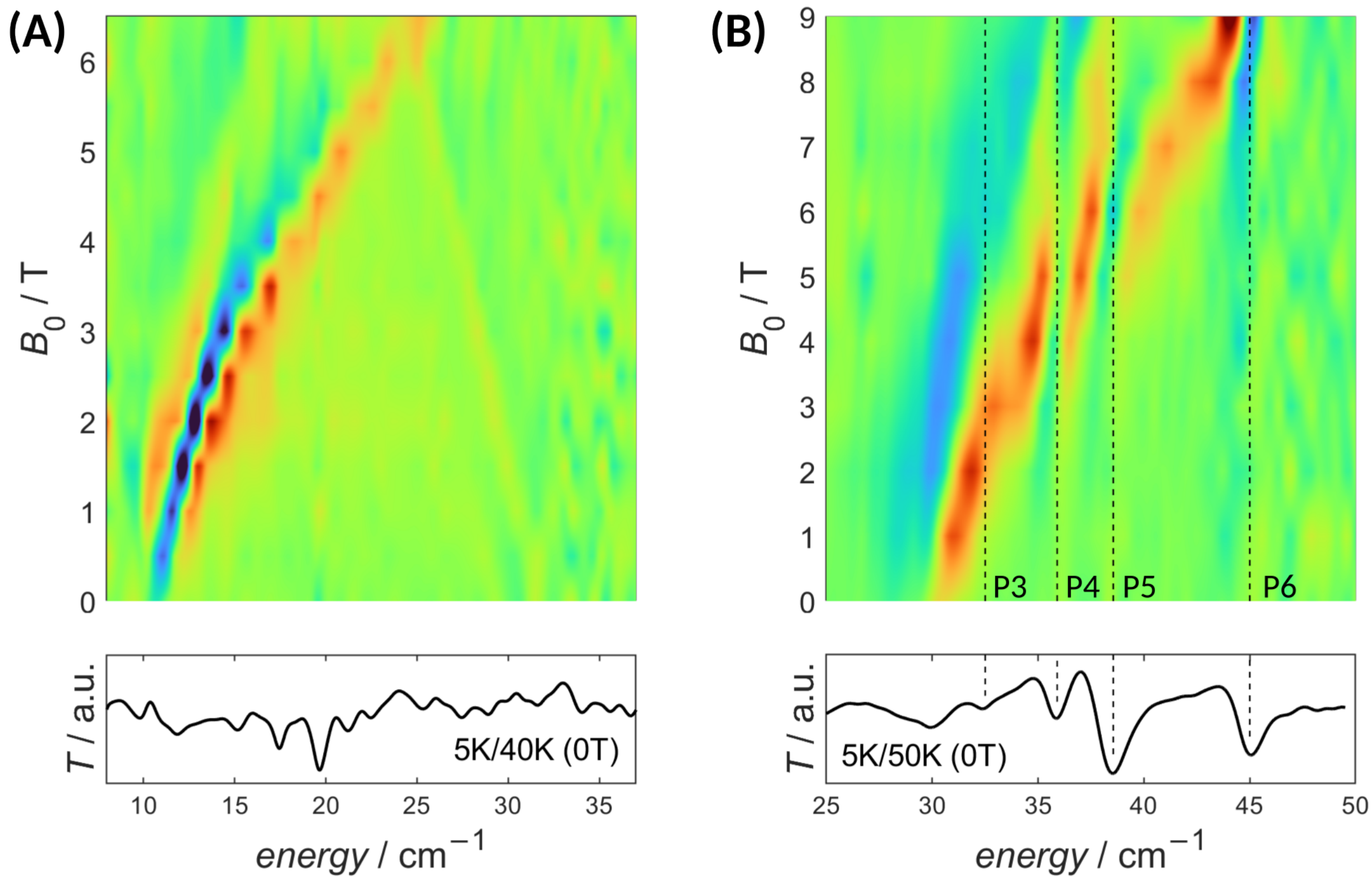

Figure S1: Contour plots of the MDS of compound **1** (A) and **2** (B). Phonon spectra are represented as temperature-division spectrum (T : relative transmittance). At the positions of the phonons in (dotted lines) avoided crossings are visible indicating spin-phonon coupling. Note that the ripple-like features in the intensity of **1** are due to the rendering of the experimental spectra, which had been taken in steps of 0.5 T (A) and 1 T (B), into the contour plot.

Table S1: Best parameters for the numerical simulation of the THz EPR spectra of **1** and **2** based on a pure spin Hamiltonian approach (Eq. 1).

Parameter	[Fe(3−Metpz)$_2$] (**1**)	[Fe(bpz)$_2$(DiMephen)] (**2**)
g_x, g_y, g_z	2.2(1), 2.3(1), 2.00(5)	2.4(2), 2.45(5), 2.0(5)
D/cm^{-1}	10.5(1)	15.41(5)
E/D	0.035(5)	0.250(2)
Gaussian linewidth Γ_G/cm^{-1}	—	1.2
Lorentzian linewidth Γ_L/cm^{-1}	—	1.5
D-Strain $(\Gamma_\mathrm{D}^x, \Gamma_\mathrm{D}^y, \Gamma_\mathrm{D}^z)$/cm^{-1}	0.35, 0.5, 0.1	—

Note that for the simulation of the spectra of **1** an anisotropic linewidth, i. e. D-Strain, was nessesary to reproduce the relative intensities of the transitions in sets **A** and **B**.

Spin-Phonon Coupling

Simulation of the MDS of complex **2** was based on an extended spin-Hamiltonian that includes the first order spin-phonon coupling.[1]

$$\hat{H}_{\mathrm{tot}} = \hat{H}_S + \hat{H}_{\mathrm{Ph}} + \hat{H}_{S-\mathrm{Ph}}$$

$$\hat{H}_{\mathrm{Ph}} = \sum_{\alpha}^{N} \hbar\omega_\alpha (\hat{n}_\alpha + \frac{1}{2})$$

$$\hat{H}_{S-\mathrm{Ph}} = \sum_{\alpha}^{N} \frac{\partial \hat{H}_S}{\partial Q_\alpha} \hat{Q}_\alpha$$

Here, $\hat{H}_S$ is the spin-Hamiltonian in Eq. 1, $\hat{H}_{\mathrm{Ph}}$ is the Hamiltonian of the four included phonons and $\hat{H}_{S-\mathrm{Ph}}$ is the spin-phonon coupling Hamiltonian. To reproduce the spectra, beside the variation of D and E with the normal coordinates ($\mathrm{d}D/\mathrm{d}Q$ and $\mathrm{d}E/\mathrm{d}Q$), also the variation of the ZFS tensor principal axes under vibration was included ($\mathrm{d}D_{xy}/\mathrm{d}Q$, $\mathrm{d}D_{xz}/\mathrm{d}Q$ and $\mathrm{d}D_{yz}/\mathrm{d}Q$). The energies and relative intensities of the uncoupled phonons with respect to the magnetic transitions were determined directly from the raw THz EPR absorbance spectrum recorded at 5 K (Fig. S3). The simulation yielded the parameters summarized in Table S2. As the simulation includes a total of 59 parameters, thus it is overparameterized, and the magnitude of the determined spin-phonon coupling should be regarded as an estimate of the order of magnitude.

Table S2: Spin phonon coupling parameters for phonons P3 - P6 (top to bottom).

Vibrational frequency [cm^{-1}]	32.30
Magnitude of vib. transition	1.00
Vib. trans. moment angle theta in ZFS PAS (0-180)	0.00
Vib. trans. moment angle phi in ZFS PAS (0-360)	0.00
Spin phonon coupling (dD/dQ) D' [cm^{-1}]	0.000
Spin phonon coupling (dE/dQ) E' [cm^{-1}]	0.000
Spin phonon coupling (dDxy/dQ)Dxy'[cm^{-1}]	0.500
Spin phonon coupling (dDxz/dQ)Dxz'[cm^{-1}]	0.000
Spin phonon coupling (dDyz/dQ)Dyz'[cm^{-1}]	0.000
Vibrational frequency [cm^{-1}]	35.85
Magnitude of vib. transition	3.00
Vib. trans. moment angle theta in ZFS PAS (0-180)	0.00
Vib. trans. moment angle phi in ZFS PAS (0-360)	0.00
Spin phonon coupling (dD/dQ) D' [cm^{-1}]	0.000
Spin phonon coupling (dE/dQ) E' [cm^{-1}]	0.300
Spin phonon coupling (dDxy/dQ)Dxy'[cm^{-1}]	0.300
Spin phonon coupling (dDxz/dQ)Dxz'[cm^{-1}]	0.300
Spin phonon coupling (dDyz/dQ)Dyz'[cm^{-1}]	0.000
Vibrational frequency [cm^{-1}]	38.50
Magnitude of vib. transition	5.00
Vib. trans. moment angle theta in ZFS PAS (0-180)	0.00
Vib. trans. moment angle phi in ZFS PAS (0-360)	0.00
Spin phonon coupling (dD/dQ) D' [cm^{-1}]	0.000
Spin phonon coupling (dE/dQ) E' [cm^{-1}]	0.000
Spin phonon coupling (dDxy/dQ)Dxy'[cm^{-1}]	0.500
Spin phonon coupling (dDxz/dQ)Dxz'[cm^{-1}]	0.000
Spin phonon coupling (dDyz/dQ)Dyz'[cm^{-1}]	0.000
Vibrational frequency [cm^{-1}]	45.00
Magnitude of vib. transition	4.00
Vib. trans. moment angle theta in ZFS PAS (0-180)	0.00
Vib. trans. moment angle phi in ZFS PAS (0-360)	0.00
Spin phonon coupling (dD/dQ) D' [cm^{-1}]	0.000
Spin phonon coupling (dE/dQ) E' [cm^{-1}]	0.000
Spin phonon coupling (dDxy/dQ)Dxy'[cm^{-1}]	0.300
Spin phonon coupling (dDxz/dQ)Dxz'[cm^{-1}]	0.000
Spin phonon coupling (dDyz/dQ)Dyz'[cm^{-1}]	0.000

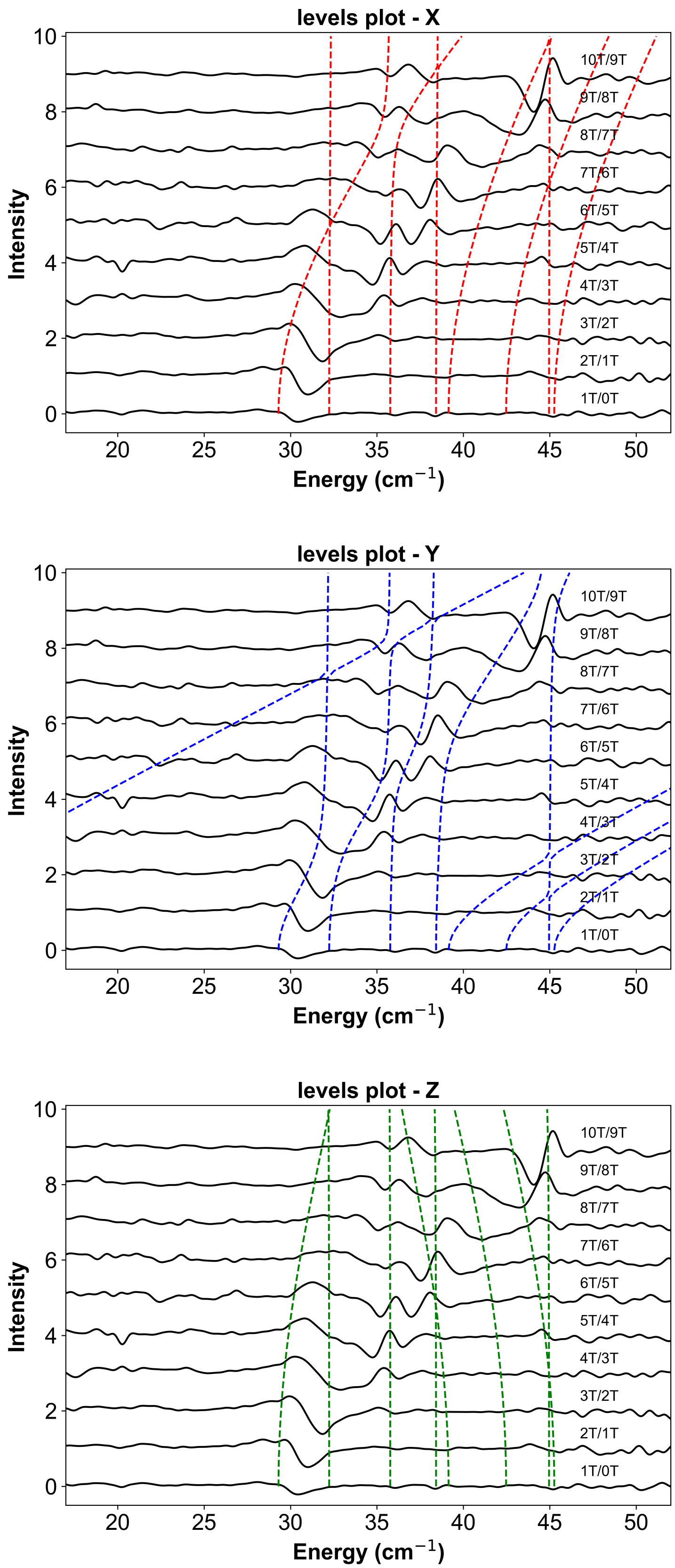

Figure S2: Simulated field-dependent transition energies for magnetic field applied along the ZFS principal axes, based on the spin-phonon coupling extended spin-Hamiltonian model superimposed on the experimental MDS. Note that the MDS were obtained by dividing spectra recorded at higher field by spectra recorded at lower field as indicated in the figures.

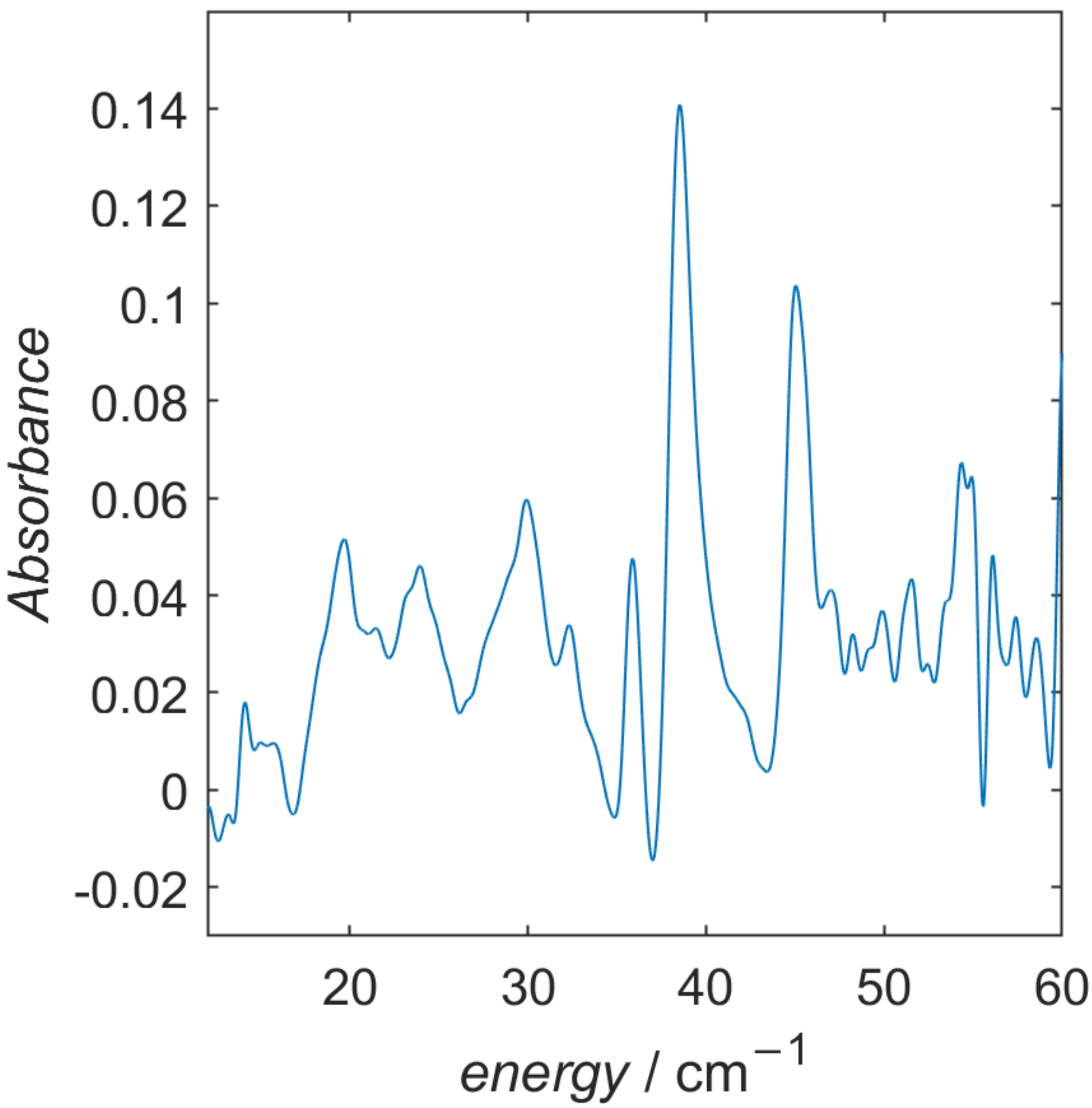

Figure S3: Absorbance spectrum of **2** referenced to a pure HDPE pellet.

References

(1) Chen, H.; van Gastel, M.; Schnegg, A.; Neese, F. Bridging Vibrations and Spins: Mode-Resolved Spin-Phonon Coupling Revealed through THz EPR/Magnetic IR Simulation. *J. Phys. Chem. A* **2026**, *130*, 1486–1500.
